# Physicians advice, parental practice and adherence to doctor’s advice: an original survey on infant feeding

**DOI:** 10.1186/s12887-019-1697-y

**Published:** 2019-09-04

**Authors:** Jean-Pierre Chouraqui, Bérénice Delmas, Marine Le Bris, Marc Bellaiche, Camille Jung, Thierry Hanh

**Affiliations:** 1 Paediatric Nutrition and Gastroenterology, Division of Pediatrics, Woman, Mother and Child Department, Centre Hospitalier Universitaire Vaudois (CHUV), 21 rue du Bugnon, 1011 Lausanne, Switzerland; 2 Nestlé, Département Médical et Nutrition Infantile, 7 Boulevard Pierre Carle, BP 900 Noisiel, France; 3 Service de Gastroentérologie, Mucoviscidose et Nutrition Pédiatriques, Hôpital Robert Debré, 48 Boulevard Serurier, 75935 Paris, France; 4 Centre de Recherche Clinique, CHI Creteil, 40 Avenue de Verdun, 94000 Creteil, France

**Keywords:** Adherence to physician’s advice, Breastfeeding, Complementary foods, Evaluation of practices, Functional gastrointestinal disorders, Infant feeding practices, Infant formula

## Abstract

**Abstract:**

Background: Despite several years of guidance on infant feeding, there has been only a slight change in consumer compliance. Therefore, this study aimed to analyse parents feeding practices, explore physicians’ advice about infant feeding and subsequent parent’s adherence to advice.

**Methods:**

A multicentric cross-sectional qualitative and descriptive self-report online study was conducted in volunteers to participate in the study.

**Results:**

Fifty-four physicians (paediatricians and general practitioners) and 600 parents of infants were recruited. Of the infants, 20.2% presented at inclusion with at least one type of functional gastrointestinal disorder. The breastfeeding prevalence was quite low (37.3%). The main initial deviance from guidelines said they observed in infant feeding was the early use of cow’s milk. More than two-thirds of infants older than 8 months were drinking cow’s milk. The introduction of solid foods was globally in line with recommendations. Most physicians gave advice about the different aspects of infant feeding but were seeking more information, as did the parents. A discrepancy between the physicians’ statements and the parents’ perceptions was observed. However the majority (95.4%) of parents reported that they followed totally or partially the advice received, especially by abandoning subsequently the use of cow’s milk in favour of a formula. The main reason for not adhering to the advice was that they did not consider it suitable for their infant and they preferred to rely on their feelings or recommendations from familiars.

**Conclusions:**

This survey provides good insights into parents’ infant feeding practices together with the advice given by their doctor. The gap between practices and current guidelines is notable only for breast-feeding and use of formula. Despite several guidelines professionals and parents seek nutrition information. It highlights the need to deliver consistent, relevant, and less confusing messages about infant feeding.

**Electronic supplementary material:**

The online version of this article (10.1186/s12887-019-1697-y) contains supplementary material, which is available to authorized users.

## Introduction

Infant feeding is an important determinant of both growth and development and poses medium and long-term risks for developing non-communicable chronic diseases [1]. Different guidelines for feeding practices and nutrient requirements have been released over the years [2–8]. However, the general observation is that parents’ dietary practices are incongruent with the guidelines [9–16]. A recent survey of 1184 families in France highlighted the low prevalence and duration of breastfeeding and the early abandonment of infant formulas for the use of cow’s milk, while the introduction of solid foods was done within the recommended time range [16]. While many factors may account for this gap [13], practitioners have a responsibility to provide evidence that is based on the best practice advice. Therefore, the aims of the present survey were: first to analyse the parents’ prior practices in their infant’s feeding, and to describe the subsequent physicians’ advice on this feeding, and second to assess the parents’ adherence to physician advice.

## Materials and methods

A multicentric cross-sectional qualitative and descriptive online study was conducted in mainland France from 5 February to 31 December 2016, according to the ethical standards.

### Participants

All paediatricians and general practitioners (GPs) who were known to be regularly engaged in infant primary ambulatory care were invited by mail or email to participate in the study. Once agreed, they received a username and personal password to access a dedicated secure website (Optinutri.fr). Each practitioner had to invite parents of a first healthy infant aged 1 to 12 months to participate, provided they were fluent in French and able to use the Internet. The infants were divided into five age groups corresponding to the usual ages that the diet is modified: 1–2, 3–4, 5–6, 8–9, and 11–12 months (infants aged 7 or 10 months were excluded). After receiving verbal and written explanations of the purpose and content of the research parents gave first verbal consent and provided their e-mail address and telephone number, which were registered and encrypted on the website using a stream cipher (RC4, RSA Security LLC, MA, USA), and email and text messages were immediately sent to the parents: one with the link to the parent questionnaire and another with their personal access code. Parents confirmed their consent online when they first connected.

### Study design

After baseline data were collected online, the physician interviewed the parents and examined the infant to complete the questionnaire. A remuneration equivalent to the price of an infant consultation was awarded for this work. Two weeks after this visit, the parents were contacted by email and text message and asked to access the website and complete their questionnaire, which was inaccessible to the physician.

### Questionnaires

Online questionnaires were developed by the investigators using C# (ASP.net) framework (version 2.0; Microsoft Corporation, WA, USA) together with Visual Studio software 2008 (Microsoft Corporation, Redmond, WA, USA). Data storage was externally managed using SQL server software (version 2008 R2; Microsoft Corporation, Redmond, WA, USA).

Both questionnaires were structured and consisted of multiple choice, yes/no, and open-ended questions (103 for physicians and 44 for parents) grouped into predefined sets. Physician questionnaires included (Additional file 1*)*: birth and current infant characteristics; parents’ allergy history, as defined by Kjellman [17]; functional gastrointestinal disorders (FGID) according to the Rome III criteria [18]; infant feeding practices since birth and currently, based on a 24 h recall; dietary advice given in relation to any breastfeeding, use of infant formula, the introduction of solid foods, or a possible FGID; and the time spent providing this advice. Parent questionnaires (Additional file 2) asked about infant feeding at follow-up, adherence to the doctor’s advice, observance in their daily practice, and their feelings about the time devoted to this advice. After questionnaire completion, all parent identifiers were deleted from the data. At the end of the survey, the database was frozen after backup and access to the survey website was deactivated.

### Data analysis

Results were given as the number, percentage, and median with the interquartile range (Q1–Q3). The Stat Process of SAS 9.2 (SAS Institute, Cary, NC, USA) was used for statistical analyses. Pearson’s chi-squared or Fisher’s exact tests with the Monte Carlo algorithm were used for the qualitative categorical and binary data, the Kruskal–Wallis test was used as a non-parametric test for the quantitative data. To compare doctor and parent’s perception on the same item McNemar’s test was used for the paired nominal data, and Cohen’s kappa coefficient (κ) measured inter-rater agreement for the qualitative (categorical) items. A significance level of 0.05 was used, with a 95% confidence interval.

## Results

Of the contacted physicians, 159 (1.2%) answered the invitation; 54 (34%) physicians, including 31 paediatricians and 23 GPs, agreed to the protocol and conducted the study. The paediatricians included 461 infants (76.8%) and the GPs included 139 infants (23.2%). Three infants were excluded because they did not meet the inclusion criteria. The mother was the usual respondent (93.4%).

### Data at inclusion

#### Infants’ characteristics

Infants’ characteristics are shown in Table 1. A history of allergic disease was noted in 32.8% of the infants’ parents (mother: 14.9%; father: 12.4%; both: 5.5%).

**Table 1:** Infants’ characteristics at inclusion, including gestational age, birth weight and length, and current age, weight, and length, given as the median with the interquartile range (Quartile 1 to Quartile 3)

	Age group (months)					
	1–2	3–4	5–6	8–9	11–12	Total *N* = 600
Birth data						
Number	150	132	146	90	79	597
Number (%) of Preterm infants	7 (4.7%)	17 (12.9%)	13 (8.9%)	6 (6.7%)	7 (8.9%)	50 (8.4%)
Birth weight (kg)	3.31 [3.05–3.57]	3.28 [2.91–3.65]	3.27 [3.00–3.50]	3.27 [2.95–3.60]	3.30r [3.00–3.55]	3.29 [2.99–3.57]
Birth length (cm)	49.70 [48.00–51.00]	50.00 [48.00–51.00]	50.00 [48.00–51.00]	49.00 [48.00–51.00]	49.50 [48.00–51.00]	50.00 [48.00–51.00]
Inclusion data						
Number	151	132	147	90	80	600
Sex ratio M/F	1.40	1.28	1.10	0.91	1.22	1.19
Age (weeks)	9.00 [4.00–9.00]	17.00 [14.00–18.00]	24.00 [23.00–26.00]	39.00 [37.00–40.00]	50.00 [48.00–52.00]	22.00 [12.50–37.00]
Weight (kg)	4.90 [4.15–5.40]	6.51 [6.11–7.19]	7.60 [7.00–8.30]	8.76 [8.20–9.22]	9.70 [8.72–10.20]	7.20 [5.78–8.50]
Length (cm)	56.00 [54.00–58.00]	63.00 [60.25–64.50]	66.00 [65.00–68.00]	71.25 [69.00–73.00]	75.00 [73.00–77.00]	65.00 [59.75–70.00]

#### Functional gastrointestinal disorders

**Fig. 1:**
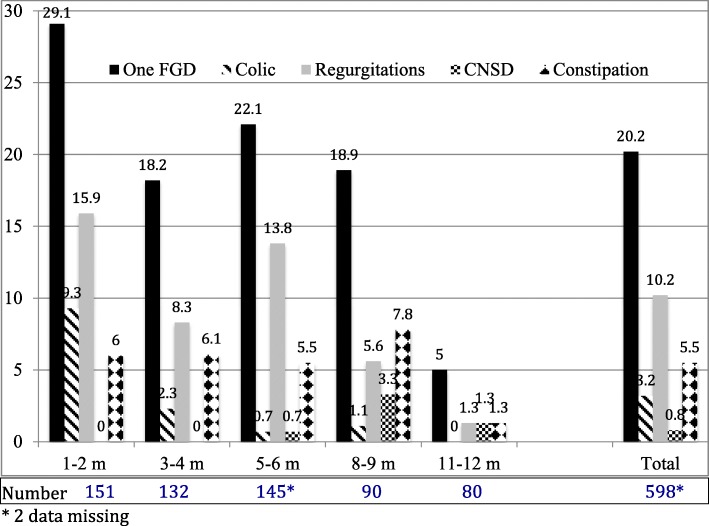
Prevalence (percentage) of functional gastrointestinal disorders (FGID) in the infants (*n* = 598) at inclusion, which included at least one type of FGID, colic, regurgitation, chronic non-specific diarrhoea (CNSD), and constipation

Figure 1 presents the prevalence of FGID, which was higher in the GPs’ practices (29.5% vs 17.4%, *p* = 0.003). Simultaneous regurgitation and crying were noted in 7.8% of the infants, and constipation together with another FGID were present in 4.9% of the infants. Infants with regurgitation had 2.00 (1.00–4.00) regurgitation episodes per day (maximum 8.00). Excessive crying mainly occurred in the evening (57.9%) and rarely during the night (15.8%). Constipated infants had 4.00 (4.00–7.00) movements per week (minimum 1.00). The stools were described as hard in 60.7% of the infants and defecation was painful in 45.5%.

#### Feeding practices

**Fig. 2:**
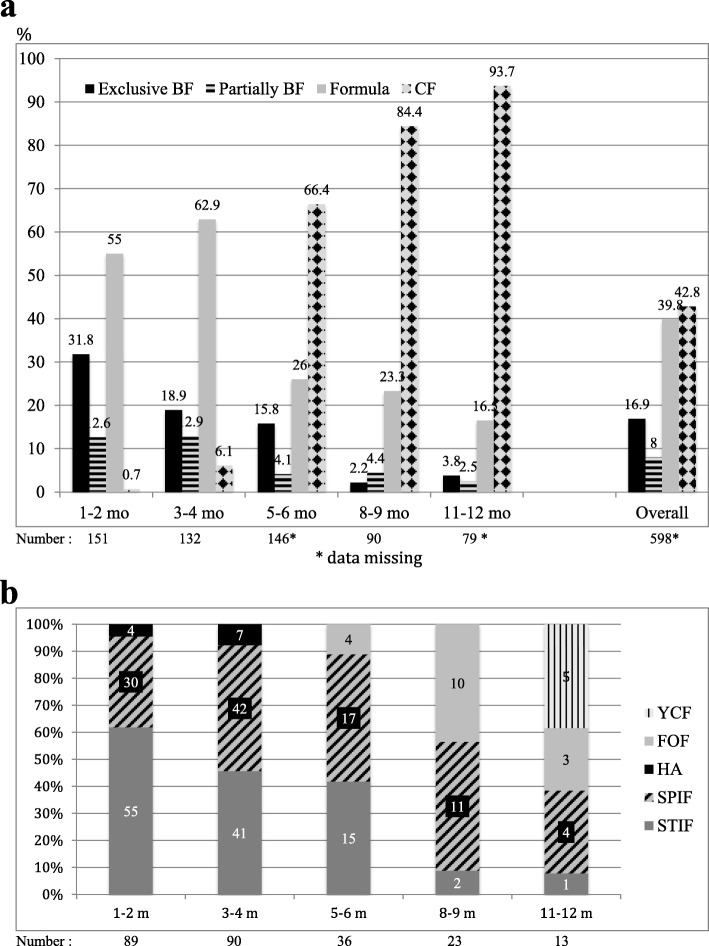
Feeding practices at inclusion: (**a**) percentage of infants in the different age groups who were either exclusively breastfed (BF), partially breastfed, or formula fed, and those who consumed complementary foods (CF) including cow’s milk and excluding formula and water (*n* = 598) [2, 6], and (**b**) the prevalence of the consumption of each category of formula in formula-fed infants, when indicated (*n* = 251). The types are identified as standard infant formula (STIF), specific infant formula (SPIF), hypoallergenic formula (HA), follow-on formula (FOF), and young child formula (YCF). The data labels represent the number of infants

The prevalence of breastfeeding at the time of inclusion is depicted in Fig. 2a. No difference was observed between practitioners except in the 1–2 months age group (37.3% of paediatricians vs 20.4% of GPs, *p* = 0.04). Information was obtained about breastfeeding at birth for 63.7% of the current non-breastfed infants and there was a positive result in 21 infants. The subsequent estimated overall prevalence of breastfeeding at birth was 37.3%. Problems with breastfeeding were reported by 16.7% of the breastfeeding mothers, which included ways to enhance lactation, engorgement or cracked nipples, having infants with a ‘gourmet’ or ‘rester’ feeding style, and/or planning their return to work. Overall, 286 (47.8%) infants consumed a formula either exclusively (83.2%) or together with breastfeeding. After 2 months of age, an increasing proportion of infants were fed cow’s milk (Table 2). The type of formula used by 87.8% of the infants is detailed in Fig. 2b; there was no difference between physicians. More than 40% (115) were fed a specific infant formula designed for either FGID (regurgitations: 36; colic: 3; constipation: 15; satiety: 8; miscellaneous: 31), low birth weight (4), prevention of an allergy (HA formula: 11), or suspected cow’s milk allergy (7).

**Table 2:** Comparison of milk diet practices between inclusion (I) and follow up (F). Results are given as number and percentage

Age group	1–2 mo		3–4 mo		5–6 mo		8–9 mo		11–12 mo		Overall	
	I	F	I	F	I	F	I	F	I	F	I	F
Number	151	105	132	102	146	111	90	63	79	56	598	437
Exclusive BF	48 (31.8%)	28 (26.7%)	25 (18.9%)	17 (16.7%)	23 (15.8%)	19 (17.1%)	2 (2.2%)	5 (7.9%)	3 (3.8%)	3 (5.4%)	101 (16.9%)	72 (16.5%)
Partial BF	19 (12.6%)	15 (14.3%)	17 (12.9%)	15 (14.7%)	6 (4.1%)	9 (8.1%)	4 (4.4%)	3 (4.8%)	2 (2.5%)	4 (7.1%)	48 (8.0%)	46 (10.5%)
Formula	83 (55.0%)	61 (58.1%)	82 (62.1%)	69 (67.6%)	38 (26.0%)	80 (72.1%)	21 (23.3%)	53 (84.1%)	13 (16.5%)	46 (82.1%)	238 (39.8%)	309 (70.7%)
CM	1 (0.7%)	1 (1.0%)	8 (6.1%)	1 (1.0%)	79 (54.1%)	2 (1.8%)	63 (70%)	0 (0.0%)	61 (77.2%)	2 (3.6%)	211 (38.5%)	6 (1.4%)
p	NS		NS		< 0.0001		< 0.0001		< 0.0001		< 0.0001	

*BF* breast_feeding, *CM* Cow’s milk, *mo* months

### Medical advice about feeding

**Fig. 3:**
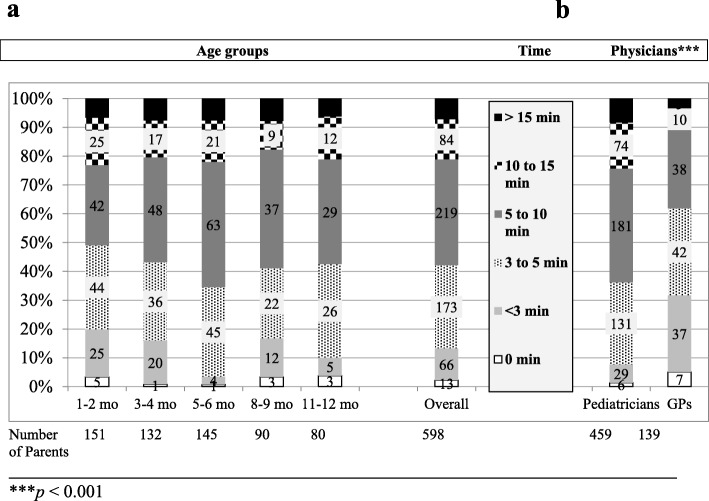
Time (minutes; min) dedicated to feeding advice: (**a**) by age group (months; mo) and (**b**) by physician type (paediatricians or general practitioners (GPs)). The results are given as the percentage of parents to whom time was devoted in each time range. The numbers in the labels represent the number of parents

Medical advice given to 97.8% of the parents and the time spent doing so are presented in Fig. 3a; the time spent was longer for paediatricians (Fig. 3b). Time spent was considered insufficient by 8.7% of the physicians, with paediatricians wishing for 15 min (10–20, *n* = 38) and GPs for 10 min (5–15, *n* = 13) (*p* = 0.001).

Breastfeeding advice (Table 3) did not differ between physicians but paediatricians more frequently gave advice about using infant formula (107 vs 16, *p* = 0.01). Of the 121 recommendations given for choosing a formula, 43.8% concerned a specific infant formula (i.e., thickened formula 47.2%, formula for constipation 20.8%, and formula for colic 15.1%). Advice about introduction of solid foods was given to 59% of parents (Table 3), and given more frequently by paediatricians (62.3% vs 48.2%, *p* = 0.002).

**Table 3:** Physicians advice topics as given to breastfed (BF) or formula fed infants’ parents, and about solid foods. Topics given as number and (percentage) of advices

**Breastfed infants** (*n* = 149)	
Advices given	100 (67.1%)
Topics (% of advices)	
Feeding frequency	57 (57.0%)
Duration of each feeding	36 (36.0%)
Benefits of BF	32 (32.0%)
Intended weaning	45 (45.0%)
Maternal diet	26 (26.0%)
Consequences of smoking, taking alcohol, drugs or medications.	19 (19.0%)
**Formula fed infants** (*n* = 286)	
Advices given	123 (43.0%)
Topics (% of advices)	
Kind of formula	122 (98.4%)
Volume/bottle	108 (87.8%)
Feeding frequency	89 (72.4%)
Volume/day	78 (63.4%)
**Solid foods** (*n* = 598)	
Advices given	353 (59%)
Topics (% of advices)	
Age of introduction of solid foods	223 (63.2%)
Order of introduction	196 (55.5%)
Modes of administration	194 (54.5%)
Progressive texture	256 (72.5%)
Quantity of each type of food	294 (83.3%)
Home-made or specific baby-food	157 (44.5%)
Varied diet	266 (75.4%)
Sharing meal with family	94 (26.6%)
Behavior in case of food refusal	208 (58.9%)
Age of introduction of Gluten	166 (47%)

The recommended age for introduction of solid foods was between 4 and 6 months in 85.2% of cases. Cooked vegetables were the first category introduced (76%), followed by cooked fruits (5.6%), or both (14.8%), and infants’ cereals were recommended in only 1.5% of cases. Spoon-feeding was the recommended mode of introduction in most cases (89.2%). Advice about quantities and gradual increases was more common for vegetables or fruit (84.4%) than for meat or fish (57.1%). Of 157 pieces of advice about the type of food to give, 40.8% favoured homemade meals, 13.4% specific commercial baby foods, and 45.9% suggested both. For meal preparation, 86.3% of the advice recommended limiting added salt, 69.3% was about limiting added sucrose, 49.5% was about cooking method, and 25.9% was related to washing vegetables and fruits. In the case of food refusal, parents were advised ‘do not force’ (60.6% of 208 recommendations) or ‘offer the disliked food repeatedly several times in the following days’ (54.3%).

When gluten introduction was addressed, the main advice (93.6%) concerned optimal age, which was between 4 and 6 months in 31% of cases, 4 and 7 months in 23.9%, and after 6 months in 44.5%. The mode of introducing gluten was addressed in 88% of cases, by adding cereals to the bottle in 58.9%, and by gradually increasing quantity in 80.7%. Postponing introduction of solid foods was recommended for 27.7% of infants with a parental allergy history, mainly for tropical fruits (61.3%) or nuts (23.1%).

There were differences in the number of paediatricians and GPs providing advice relating to introduction of solid foods mode (*p* < 0.001), food texture (77.6% vs 50.7%, *p* < 0.001), food quantity (73.4% vs 46.3%, *p* = 0.046), response to food refusal (64.3% vs 36.4%, *p* < 0.001), and introducing gluten (50.3% vs 32.8%, *p* = 0.01).

Among the 54 physicians, 49 (90.7%) wanted more information about infant feeding topics listed in Table 4; 64% preferred access via a journal, 44% via a newsletter, 38% via the Internet, 20% via a company document, 8% via a smartphone app, and some chose several options.

**Table 4:** Topics of interest about which physicians would like to obtain more information (*n* = 49) shown as the number and percentage of requests

Topics	Number of requests (%) for the information	Number of requests (%) first cited
Scientific societies’ guidelines	27 (50.0)	12 (24.5)
Diet of infants with FGID	27 (50.0)	2 (4.1)
Nutrient requirement	26 (46.3)	12 (24.5)
Prevention of allergy	21 (38.9)	8 (16.3)
Protein requirement	18 (33.3)	4 (8.2)
Food introduction/age	18 (33.3)	2 (4.1)
Textures/age	16 (29.6)	1 (2.0)
Gluten introduction	14 (25.9)	1 (2.0)
Treatment of food allergy	14 (25.9)	2 (4.1)
Quantities of milk/age	9 (16.7)	4 (8.2)
Weaning	7 (13.0)	1 (2.0)

*FGID* functional gastrointestinal disorders

### Parents’ perceptions and adherence to medical advice

Four hundred and thirty-seven parents (72.8%) completed their questionnaire an average of 16 days (14–26) after the initial visit.

#### Parents’ perceptions

Among these, 434 (99.3%) answered the questions about medical advice, with 414 (95.4%) reporting they received one or more pieces of dietary advice and 43% obtaining a brochure that mainly explained the introduction of solid foods. Overall, the parents’ statements about receiving feeding advice were discordant with the physicians’ statements (*p* < 0.0001, κ = 0.11); 14.3% claimed to have received advice that was not reported as given by the physician. This discrepancy was significant in the groups with infants under 5 months. The comparison of the time spent in consultation reported by the parents and that declared by the physician was concordant in 28.9% of the parents, whereas 33.6% thought that this time was longer and 37.4% thought that it was shorter. Of the parents, 84.4% reported that the consultation time was sufficient versus 91.3% of the physicians, and there was no concordance between the answers (*p* < 0.0001, κ = 0.079).

#### Parents’ adherence

When all advice was combined, 4.6% of the counselled parents said they had not followed the advice given so far, 8.7% had followed it partially, 45.3% had followed it mostly, and 41.3% had followed it totally. The parents of younger infants were the most adherent (*p* < 0.001). In descending order, the five most followed pieces of advice were the age for introduction of solid foods, the order in which to introduce the solid foods categories, the choice of formula, food quantity at each age, and food category amounts. About 75% parents reported that advice they received resulted in improved condition in FGID infants. The most frequent reason parents gave for non-adherence was that it was not suitable for their infant (54.4% of the 22 pieces of infant formula advice not followed and 42.9% of the 91 pieces of introduction of solid foods advice not followed). The second reason was personal choice followed by conflicting advice from family and friends. The five least followed pieces of advice were about weaning, the amount of formula, the advantage of homemade food, the advantage of specific baby food, and the age for introducing gluten.

#### Dietary practices at the follow-up

Following the consultation, the consumption of cow’s milk by infants older than 5 months was significantly reduced, replaced by more frequent use of formula (Table 2).

## Discussion

Since few studies [9–12, 15, 16, 19–23] have conducted similar investigations, this pilot study provides important data on differences between professionals’ perceptions and parents’ perceptions and subsequent behaviour in regard to infant feeding, and highlights a number of key issues important to developing future guidelines. Except for one earlier study [19] the current study is the only one to recruit both paediatricians and GPs.

Although it is not certain that the Rome III criteria were applied strictly, the prevalence of FGID in our sample was much lower than previously reported [24, 25]. The GPs higher proportion of FGID infants may be due to patient and family differences but may also be a result of poor knowledge of the criteria [18]. The prevalence of parental atopic history is higher than that originally described in one study (14.3%) [17], but similar or even lower than in more recent studies (27.2 and 43.2%) [26, 27].

This survey recognised that the majority of physicians and parents were aware of infant feeding methods. However, initial dietary practices in our sample were far from optimal regarding breastfeeding and the preference for infant formula instead of cow’s milk [2–6]. No information was collected about what may have influenced these practices. The prevalence of breastfeeding is generally reported as low in France, [16, 28, 29]. A surprisingly large proportion of non-breastfed infants in our sample had already consumed cow’s milk, which was higher than that reported in the Nutri-Bébé study (5.1%) [16], and less than half of the infants consumed a formula. Of note is the small number of children who were fed HA formula considering the observed prevalence of family allergy history, but at-risk infants were not identified among the breastfed infants. This contrasts with the results of the AllerNaiss study reporting a screening strategy for allergy history in 59% of 387 French maternity wards and a subsequent prescription of HA formula, in the absence of breastfeeding in 90% of the wards [30].

The majority of parents adhered to the guidelines when introducing solid foods. No infant younger than 5 months consumed them. In the Nutri-Bébé study, the mean age for the introduction of solid foods was 5.4 ± 2.1 months [16]. These results are more encouraging than those reported by other researchers [9, 11, 19]. Of note, however, is the persistence of postponing introducing some specific foods in case of parental allergy history. Information given about texture was more frequent than previously reported [22].

Most of the physicians spent time giving advice. Overall, this advice was in line with the guidelines, whereas in a study conducted in southern France, only 65% of paediatricians (*n* = 50) or paediatric residents (*n* = 34) gave such advice [21]. Physicians would like to obtain more information about infant feeding practices, which highlights the gap between the publication of scientific guidelines and professionals’ perception.

In the follow-up, the parents’ response rate was high and very few said they were not observing the advice. As a result, the main deviance initially observed, the early consumption of cow’s milk, was corrected. Among non-compliant parents, the reason given was lack of confidence in their doctor and a preference for advice from family and friends or other environmental sources [15, 19, 20]. It is astonishing to note that the advantages of homemade food or specific baby food and the age for introducing gluten were among the least followed pieces of advice. There is a large discrepancy globally between physicians’ statements and parents’ perceptions. While reported compliance with current infant feeding guidelines was not terribly poor, there is nevertheless a long way to go before the behaviour perfectly matches the guidelines. As proposed by others, the parents’ and physicians’ interest in nutrition and health, coupled with their confusion, suggests that there are shortcomings in current nutrition communication and that there are important opportunities to improve dietary guidance [13, 31].

Our results should be viewed in the context of the study’s limitations. The number of participating physicians was relatively small, but they recruited a larger sample of parents than in other studies [9, 15, 20, 23]. We acknowledge that our survey recruited a selected and demographically biased sample of parents who were mostly educated and fluent in French and able to use the Internet, which is not nationally representative. Moreover, only primiparous mothers were recruited, and having more children is linearly associated with higher nutritional knowledge (*p* < 0.01) [10]. Our results are also based on self-reported practices rather than objective audit data, which may compromise reliability. Finally, while using a structured questionnaire allowed precise and comparable answers, it may also have guided the answers.

## Conclusion

While our study does not reveal a large gap between current guidelines and physicians’ and parents’ practices, a number of improvements are desirable. Given the study’s declarative aspects conclusions must be interpreted with caution. However, an important point was raised—despite the existence of several published guidelines, some professionals and parents seek nutrition information while others ignore the recommendations, emphasising the need to review strategies for promoting good nutrition. Providing consistent and relevant advice might decrease confusion and render the advice more acceptable and increasing awareness of media influences may decrease messages that conflict with scientific committees’ guidelines.

Indeed possible contradictions between opinion leaders lead to confusion, which contributes to counteracting nutrition counselling in general and lead parents to consider scientists really do not know what foods are good for their child. But what matters is that dietary advice must clearly explain the recommended behaviours and why changes have occurred so that consumers have the opportunity to develop an appreciation of how scientific knowledge evolves. Effective guidance needs to consider what really matters to parents and must promote recommendations based on foods instead of nutrients. One step could be to help parents understand why and how healthy eating can be beneficial in the short, medium and long term for their child. Nutrition messages need to be simple, clear, easy to understand, realistic, positive, and exploitable fitting with modern lifestyles without demanding a complete renunciation of habits. An objective audit of medical practices is needed but may be difficult to achieve.

## Additional files

Additional file 1: Physician questionnaires including birth and current infant characteristics; parents’ allergy history; functional gastrointestinal disorders; infant feeding practices since birth and currently; dietary advice; time spent providing the advice. (PDF 181 kb)

Additional file 2: Parent questionnaires including infant feeding at follow-up, adherence to the doctor’s advice, observance in their daily practice, and their feelings about the time devoted by the physician to the advice. (DOC 92 kb)

## Data Availability

All data generated and analysed during this study are included in this published article. Nevertheless, the datasets used and/or analysed during the current study are available, on reasonable request, from the following research organization: Stat Process, 52, Boulevard Sébastopol, 75003 Paris, France. florence.mercier@statprocess.com

## Abbreviations

BF: Breast-feeding
CNSD: Chronic non-specific diarrhoea
d: day
FGID: Functional gastrointestinal disorders
FOF: Follow-on formula
GP: General practitioners
HA: Hypoallergenic formula
IF: Infant formula
ISF: Introduction of solid foods
SPIF: Specific infant formula
STIF: Standard infant formula
YCF: Young child formula
