# Supplementary material for: Physicians advice, parental practice and adherence to doctor’s advice: an original survey on infant feeding
Source: BMC Pediatr. 2019 Sep 4;19:313. doi: 10.1186/s12887-019-1697-y (PMC6724317; doi:10.1186/s12887-019-1697-y)
Supplement: Supplementary file 1 — Physician questionnaires including birth and current infant characteristics; parents’ allergy history; functional gastrointestinal disorders; infant feeding practices since birth and currently; dietary advice; time spent providing the advice. (PDF 181 kb) [file 12887_2019_1697_MOESM1_ESM.pdf]

OPTINUTRI PRACTICE SURVEY

«Nutritional advices and parental observance»

E-survey “doctor” with conditional blocks

Reference: |__|__|__|__|__|

Date of medical consultation: |__|__|/|__|__|/|__|__|

Birth information:

Birth weight: |__| kg |__|__|__|
Length at birth: |__|__| cm
Sex: male ☐ female ☐
Birth term: I__I__I weeks of amenorrhea

Information during medical consultation:

Age: |__|__|/|__|__|
Month/week
Weight: |__|__| kg |__|__|__| Length: |__|__| cm

Parents allergic history:

Father: Yes ☐ No ☐ do not know ☐
If so→: Cow’s milk protein allergy ☐ other food allergies ☐ others ☐

Mother: Yes ☐ No ☐ do not know ☐
If so → Cow’s milk protein allergy ☐ other food allergies ☐ others ☐

Current infant feeding:

- ☐ Exclusive breastfeeding
- ☐ Mixed breastfeeding
- ☐ Infant formula only
- ☐ Cow’s milk
- ☐ Solid food

Functional gastrointestinal disorders (FGID)

Does the infant have intestinal functional disorders?

Yes ☐ No ☐

If so:

Evaluation of constipation:
Is the infant constipated? Yes ☐ No ☐

If so:

Number of stools per week: |__|__|
Are the last stools hard? Yes ☐ No ☐
Pain during bowel movement: Yes ☐ No ☐
Regular use of external maneuver: Yes ☐ No ☐

What advice and/or treatments did you give?

- Advice:
  - ☐ Massage
  - ☐ Change of mineral water
  - ☐ Use of a water rich in magnesium

- Infant formula prescription (multiple choice):
  - ☐ Acidified infant formula
  - ☐ Infant formula with fibers
  - ☐ Infant formula with prebiotics
  - ☐ Infant formula with probiotics
  - ☐ Lactose-enrichied infant formula
  - ☐ Others

(specify): ……………………………………………………………………

- Prescription of medication (multiple choice):
  - ☐ Osmotic laxative
  - ☐ Lubricant laxative
  - ☐ Enema
  - ☐ Suppository
  - ☐ Others

(specify): ……………………………………………………………………

Evaluation of diarrhea:
Does the infant have diarrhea? Yes ☐ No ☐

If so

Does he have liquid stools? : Yes ☐ No ☐
If so:
- Number of liquid stools during the last 24 hours: |__|__|
- How many days have the stools been liquid? |__|__|

- Is the infant dehydrated? Yes ☐ No ☐

What treatments/infant formulas did you prescript?
☐ Prescription of medication:
☐ Oral Rehydration Solution (ORS)
☐ Others (specify): …………………………………………………………………

☐ Infant formula prescription:
☐ Usual infant formula (Drop-down list, with preselection of the last infant formula mentioned in the table of infant formula):
☐ Extensively hydrolyzed infant formula
☐ Anti-diarrhea infant formula

Evaluation of regurgitations:
Does the infant have regurgitations? Yes ☐ No ☐

If so
During the last 24 hours:
How many bottles did the infant consume?: l__l__I
How many bottles have been followed by regurgitations?: l__l__l

Evaluation of the importance of regurgitation on a scale of 0 (very low volume) to 10 (very large volume): l__l__l /10

Evaluation of the frequency of regurgitations on a scale of 0 (absence of regurgitations) to 10 (frequent regurgitations): Frequency: l__l__l /10

What advice and/or treatments did you give?
☐ Advice:
☐ Position of the infant during the meal
☐ Infant position during postprandial period
☐ To burp the baby
☐ Splitting or decreasing volume of meals
☐ Inclination of the bed
☐ Prohibition of passive smoking

☐ Infant formula prescription:
☐ Usual infant formula (Drop-down list, with preselection of the last infant formula mentioned in the table of infant formula): …………………………………………
☐ Thickened infant formula
☐ Anti-regurgitation infant formula "AR" with carob
☐ Anti-regurgitation infant formula "AR" with starch
☐ External thickener: carob
☐ External thickener: starch
☐ External thickeners: carob + starch
☐ External thickener: gelopectose

☐ Prescription of medication:

☐Drug (drop-down list)

Evaluation of colics:
Does the infant fussing and crying periods impossible to settle down? Yes ☐ No ☐

If so:
Duration of crying ≥ 3 hours per day, 3 days per week, during at least one week? Yes ☐ / No ☐

What is the most common occurrence of crying (one answer only)?
Night (between 00AM and 8AM) ☐ Day (between 8AM and 4PM) ☐ Evening (between 4Pm and 11PM) ☐

What advice and/or treatments did you give? :
☐ Advice:
- ☐ In case of breastfeeding, the mother must avoid eating dried vegetables and cabbage
- ☐ Methodes of appeasement (massage, balancing…)
- ☐ Remove causes of aerophagia

☐ Infant formula prescription:
- ☐ Usual infant formula (Drop-down list, with preselection of the last infant formula mentioned in the table of infant formula): …………………………………………
- ☐ Lactose-free infant formula
- ☐ Infant formula with L.reuteri
- ☐ Acidified infant formula
- ☐ Hypoallergenic infant formula (partially hydrolyzed)
- ☐ Extensively hydrolyzed infant formula

☐ Prescription:
- ☐ Drug (drop-down list)
- ☐ An herbal infusion

MEDICAL ADVICE

Breastfeeding:

During this medical consultation, did you give any advice/recommendations on breastfeeding for this infant?
Yes ☐ No ☐

→If so*:* you gave advice on (multiple choices):
☐ Benefits of breastfeeding on infant health?
☐ Feeding frequency?
☐ Duration of each feeding?
☐ Impact of mother's diet on breast milk?
☐ Impact of mother's lifestyle (cigarettes, alcohol, drugs…) on breast milk?
☐ Potential difficulties encountered?

: ………………………………………………………………………………………………..

Did you give advice to succeed intended weaning?
Yes ☐ No ☐

Infant formula

Specify each infant formula given to this infant/child since birth including the current one (drop-down menu for each line with number choice – number one is the first given formula) and for which medical indication (drop-down list). Add as many lines as needed:

☐ None
→otherwise:

Infant formula	Medical indication
Drop-down list by stage: manufacturer choice, brand choice and then formula choice	Drop-down medical indication list (principal and secondary)

→if at least one infant formula given:
Did you give any advice other than in the case of a functional gastrointestinal disorder?
Yes ☐ No ☐

→If so:

Did you give advice on (multiple choice):
- ☐ Feeding frequency?
- ☐ How much milk per day?
- ☐ How much milk per bottle?

To calculate the amount of milk to advise:
Did you use the Appert's formula in relation to the weight
Yes ☐ No ☐

Did you follow the indicated instructions on infant formula packaging?
Yes ☐ No ☐

Did you give any advice on the type of infant formula to use ?
Yes ☐ No ☐
→if so:
- ☐ Formula designed for breastfeeding relay
- ☐ Standard formula
- ☐ Hypoallergenic formula (HA)
- ☐ Follow on formula
- ☐ Young child formula

☐ In case of functional gastrointestinal disorders (FGID), have you advised:

☐ Thickened formula
☐ Anti-regurgitation formula
☐ Anti-colic formula
☐ Anti-diarrhea formula
☐ Formula designed for constipation
☐ Minor and combined gastrointestinal disorders

☐ Extensively hydrolyzed formula

Solid foods

At this medical consultation, did you give any advice regarding the introduction of solid foods for this infant?
Yes☐ No☐

→If so:
If so, did you give advice on:

- The appropriate age for introduction of solid foods?
Yes ☐ No ☐
→If so:

☐ Before 4 months?
☐ Between 4 and 5 months?
☐ Between 5 and 6 months?
☐ Between 4 and 6 months?
☐ After 6 months?

- Introduction of gluten?
Yes ☐ No ☐

→If so:

Did you recommend an appropriate age for introduction of gluten?
Yes ☐ No ☐
→If so:
☐ Before 4 months
☐ Between 4 and 6 months
☐ Between 4 and 7 months
☐ After 6 months

Did you explain how to do it?
Yes ☐ No ☐
→If so:
☐ By adding infant cereals in infant formula
☐ By adding infant cereals in purees
☐ By using biscuits, bread
☐ By using mixed pasta

☐ Others (specify): ……………………………………

Did you specify how much of these foods to introduce at the beginning?
Yes ☐ No ☐

- Did you specify which food to introduce first?
Yes ☐ No ☐
→If so (multiple choice):
☐ Mashed cooked vegetable
☐ Mashed cooked fruits
☐ Infant cereals
☐ Starchy foods (pasta, rice, potatoes)
☐ Meat
☐ Fish
☐ Others (specify): ……………………………………

Did you recommend any order of introduction of the different food categories?
Yes ☐ No ☐
→If so:
☐ Vegetables first
☐ Fruits first
☐ Vegetables or fruits first?
☐ Starchy foods first (pasta, rice, potatoes)
☐ Infant cereals first?
☐ Meat first?
☐ Others (specify): ……………………………………

Did you specify if it is better to introduce first the solid foods in the bottle or with a spoon?
Yes ☐ No ☐

→If so:

☐ in bottle?
☐ with spoon?
☐ No matter

Did you give advice on the texture to give to the baby?
Yes ☐ No ☐
→If so: (multiple choice):
☐ Mixed
☐ Smooth
☐ Mashed
☐ Grainy
☐ Small soft pieces
☐ Small hard pieces
☐ Others (specify)

Did you recommend quantities of each type of food categories to consume?
Yes ☐ No ☐

→ If so: (multiple choice):

- ☐ For vegetables?
- ☐ For fruits?
- ☐ For starchy foods?
- ☐ For infant cereals?
- ☐ For meat or fish?

Did you recommend food quantities in grams to the parents?
Yes ☐ No ☐

Did you recommend spoonful or household measures to the parents?
Yes ☐ No ☐

Have you recommended to parents a change in quantities with age?
Yes ☐ No ☐

If the infant is not at risk of allergy, have you recommended introducing solid foods one by one?
Yes ☐ No ☐

→If so, how often?

- ☐ One or more per day
- ☐ One every 2 days
- ☐ One every 3 days
- ☐ One per week
- ☐ Others (specify): ……………………………………

Did you recommend delaying the introduction of certain foods?
Yes ☐ No ☐

If so, which ones:
………………………………………………………………………………………

If the infant is at risk of allergy, have you recommended introducing solid foods
Yes ☐ No ☐

→if so, how often?

- ☐ One or more per day
- ☐ One every 2 days
- ☐ One every 3 days
- ☐ One per week
- ☐ Other

Did you recommend delaying the introduction of certain foods?
Yes ☐ No ☐

→if so, which ones
………………………………………………………………………………………

Did you give list of foods to proscribe?
Yes ☐ No ☐

Did you give any advice on using homemade foods or specific baby foods?

Yes ☐ No ☐

→If so:

☐ Preferably Homemade
☐ Preferably specific baby foods
☐ Both without distinction

Did you give any advice on preparing homemade purees?

Yes ☐ No ☐

If so, on:

☐ Adding salt
☐ Adding sugar
☐ Adding fats
☐ The quality of fruits and vegetables
☐ The strict washing of fruits and vegetables
☐ Cooking methods

Did you recommend:

☐ To vary the vegetables and fruits?
☐ Sharing meals with family?

If the baby refuses certain foods, did you give advices?

Yes ☐ No ☐

If so:

☐ Do not force
☐ Do not give in to the child
☐ Propose something else equivalent
☐ Propose something else
☐ Re-propose later
☐ Re-propose later several times until he appreciates it

TIME DEVOTING ON ADVICE

How much time was spent on food advices/recommendations/prescriptions during the medical consultation?

☐ No advice
☐ Between 0 and 3min
☐ Between 3 and 5min
☐ Between 5 and 10min
☐ Between 10 and 15min
☐ More than 15min

Do you consider this time sufficient?

Yes ☐ No ☐

If not: What would be the ideal time to devote on medical consultation to this subject? I__I__I minutes

NEED FOR INFORMATION

What are the topics about infant nutrition that you would like to know more about? : …………………………………………

Which types of media help you more specifically in your daily practice? (Multiple choices)

☐ Website?
→if checked, which one? …………………………………
☐ Smartphone application?
→ if checked, which one? …………………………………
☐ Newsletter
☐ Paper newsletter
