# Supplementary material for: Physicians advice, parental practice and adherence to doctor’s advice: an original survey on infant feeding
Source: BMC Pediatr. 2019 Sep 4;19:313. doi: 10.1186/s12887-019-1697-y (PMC6724317; doi:10.1186/s12887-019-1697-y)
Supplement: Supplementary file 2 — Parent questionnaires including infant feeding at follow-up, adherence to the doctor’s advice, observance in their daily practice, and their feelings about the time devoted by the physician to the advice. (DOC 92 kb) [file 12887_2019_1697_MOESM2_ESM.doc]

**OPTINUTRI PRACTICE SURVEY**

**“Nutritional advices and parental observances”**

**Parental survey – To complete 13 days after the medical consultation**

**Your doctor has given you advice about your child’s feeding, have you had the opportunity to put them in practice?**

**THIS IS AN ANONYMOUS SURVEY, YOUR DOCTOR WILL NOT HAVE ACCESS TO THESE DATA**

**Reference***:* |__|__|__|__|__|

Are you the parent who was present at the medical consultation?

**🞏** Yes

**🞏** No

Are you:

**🞏** The father

**🞏** The mother

**What is the current diet of your infant?**

**🞏** Exclusive breastfeeding

**🞏** Breastfeeding + infant formula

Specify the brand of the infant formula: Drop-down list by step: manufacturer choice, brand choice then infant formula choice

**🞏** Infant formula only

Specify the brand of the infant formula: Drop-down list by step: manufacturer choice, brand choice then infant formula choice

**🞏** Cow’s milk

**🞏** Do not drink infant formula or cow milk anymore

**🞏** Others, specify

Did your child start the solid foods? (He/she eats something other than your his/her mother milk or infant formula and water)

**🞏** Yes **🞏** No

- **During the medical consultation:**

Did you received any advice/recommendations/prescriptions from your doctor about your child’s feeding?

Yes **🞏** No **🞏**

**→If so:** Have you put these advice/recommendations/prescriptions into practice?

**🞏** Totally

**🞏** Mainly

**🞏** Partially

**🞏** Not at all

**🞏** This is for later

**→If not:** If you have not put them into practice, what is the main reason?

**🞏** You feel the advice is not suitable for your child

**🞏** Prefer to follow the recommendations of family/friends

**🞏** Prefer to follow the recommendations found by myself on the internet (websites, blogs, and forums)

**🞏** Recommended product not found in your usual shops or pharmacies

**🞏** Recommended product is too expensive

**🞏** I followed pharmacist’s recommendations

**🞏** Others (specify): …………………………………………………………………………

- **Specifically on breastfeeding:**

**🞏** Not concerned with breastfeeding

**→Otherwise**

Have you followed your doctor’s advice/recommendations in the following areas?

- Benefits of breastfeeding benefits on your infant health?
 **🞏** Yes **🞏** No **🞏** No advice given

- Impact of your diet on the quality of your milk?
 **🞏** Yes **🞏** No **🞏** No advice given

- Impact of your lifestyle (cigarette, alcohol, medication…) on your milk?
 **🞏** Yes **🞏** No **🞏** No advice given
- Feeding frequency?
 **🞏** Yes **🞏** No **🞏** No advice given

- Quantity of milk to consume?
 **🞏** Yes **🞏** No **🞏** No advice given

- Weaning (passage of breast milk to infant formula)?

**🞏** Yes **🞏** No **🞏** No advice given

- **Specifically on infant formulas:**

**🞏** Not concerned with infant formulas

**→Otherwise:**

Have you followed your doctor’s advice/recommendations in the following areas?

- The type of infant formula
 **🞏** Yes **🞏** No **🞏** No advice given

- Feeding frequency?

**🞏** Yes **🞏** No **🞏** No advice given

- The quantity of milk per day?

**🞏** Yes **🞏** No **🞏** No advice given

- The quantity of milk per bottle?

**🞏** Yes **🞏** No **🞏** No advice given

- Weaning?

**🞏** Yes **🞏** No **🞏** No advice given

**→ If at least one “No” checked:**

If you have not followed the advice/recommendations, for which reasons?

**🞏** You feel the advice is not suitable for your child

**🞏** Prefer to follow the recommendations of family/friends

**🞏** Prefer to follow the recommendations found by myself on the internet (websites, blogs, and forums)

**🞏** Recommended product not found in your usual shops or pharmacies

**🞏** Recommended product is too expensive

**🞏** I followed pharmacist’s recommendations

**🞏** others (specify) …………………………………………………………………………

- **Specifically on the solid foods:**

**🞏** Not concerned with the solid foods

**→Otherwise**

Have you followed your doctor’s advice/recommendations in the following areas?

- The appropriate age for introduction of solid foods?

**🞏** Yes **🞏** No **🞏** No advice given

- Introduction of gluten?

**🞏** Yes **🞏** No **🞏** No advice given

- The order of introduction of the different food categories?

**🞏** Yes **🞏** No **🞏** No advice given

- The quantities of each type of food categories?

**🞏** Yes **🞏** No **🞏** No advice given

- The texture of food?

**🞏** Yes **🞏** No **🞏** No advice given

- The quantity of food?

**🞏** Yes **🞏** No **🞏** No advice given

- The allergic risks and the frequency of the introduction of solid foods (one or more per day, one every two days…)

**🞏** Yes **🞏** No **🞏** No advice given

- The allergic risks and the delay of introduction of certain foods?

**🞏** Yes **🞏** No **🞏** No advice given

- Foods to proscribe?

**🞏** Yes **🞏** No **🞏** No advice given

- The interest to do homemade meals?

**🞏** Yes **🞏** No **🞏** No advice given

- How to make purees/dishes at home?

**🞏** Yes **🞏** No **🞏** No advice given

- The possible use of specific baby foods?

**🞏** Yes **🞏** No **🞏** No advice given

**→if at least one “No” checked:**

If you have not followed the advice/recommendations, for which reasons?

**🞏** You feel the advice is not suitable for your child

**🞏** Prefer to follow the recommendations of family/friends

**🞏** Prefer to follow the recommendations found by myself on the internet (websites, blogs, and forums)

**🞏** Recommended product not found in your usual shops or pharmacies

**🞏** Recommended product is too expensive

**🞏** I followed pharmacist’s recommendations

**🞏** Others (specify) …………………………………………………………………………

- **Specifically on functional gastrointestinal disorders:**

**🞏** Not concerned with functional gastrointestinal disorders

**→Otherwise**

Have you followed your doctor’s advice/recommendations in the following areas:

- wellness advice (massage, infant position…)

**🞏** Yes **🞏** No **🞏** No advice given

- The type of infant formula?

**🞏** Yes **🞏** No **🞏** No advice given

- Use of drugs ?

**🞏** Yes **🞏** No **🞏** No advice given

**→If at least one “Yes” checked:**

If so, did the advice/prescriptions improve the functional gastrointestinal disorders of your infant?

Yes **🞏** No **🞏**

**→If at least one “no” checked:**

If you have not followed the advice/recommendations, for which reasons?

**🞏** You feel the advice is not suitable for your child

**🞏** Prefer to follow the recommendations of family/friends

**🞏** Prefer to follow the recommendations found by myself on the internet (websites, blogs, and forums)

**🞏** Recommended product not found in your usual shops or pharmacies

**🞏** Recommended product is too expensive

**🞏** I followed pharmacist’s recommendations

**🞏** Others (specify) …………………………………………………………………………

- **Time devoting on advice and prescriptions during the medical consultation:**

How much time was spent on food advice/recommendations/prescriptions during the medical consultation?

🞏 No advice

🞏 Between 0 and 3min

🞏 Between 3 and 5min

🞏 Between 5 and 10min

🞏 Between 10 and 15min

🞏 More than 15min

Do you feel that the time devoted to your baby’s diet was sufficient?

Yes **🞏** No **🞏**

**→if not:**

What would be the ideal time? I__I__I minutes

Did your doctor give you any document with advice on the diet of your baby? (Breastfeeding/infant formula/introduction of solid foods)

Yes **🞏** No **🞏**

**→If so:**

🞏 Via a prescription

🞏 Paper brochure…

🞏 Website (precise which one)

This document was about:

🞏 I do not know, I have not read

🞏 Infant feeding in general

🞏 Breastfeeding

🞏 Complementary feeding

🞏 Functional gastrointestinal disorders?

🞏 Others, specify
